# Clinical effects of single femoral nerve block in combination with general anesthesia on geriatric patients receiving total knee arthroplasty

**DOI:** 10.12669/pjms.341.14071

**Published:** 2018

**Authors:** Jing Zhang, Yan Yuan, Yongjun Zhang, Ying Wang

**Affiliations:** 1 Jing Zhang, Department of Anesthesiology, The First People's Hospital of Changzhou, Changzhou 213000, P. R. China; 2 Yan Yuan, Department of Anesthesiology, The First People's Hospital of Changzhou, Changzhou 213000, P. R. China; 3 Yongjun Zhang, Department of Anesthesiology, The First People's Hospital of Changzhou, Changzhou 213000, P. R. China; 4 Ying Wang, Department of Anesthesiology, The First People's Hospital of Changzhou, Changzhou 213000, P. R. China

**Keywords:** Femoral nerve block, Geriatric, Knee arthroplasty, Laryngeal mask

## Abstract

**Objective:**

To evaluate the clinical effects of single femoral nerve block (sFNB) combined with general anesthesia on geriatric patients receiving unilateral total knee arthroplasty (UTKA).

**Methods:**

Sixty geriatric UTKA patients who were treated in The First People's Hospital of Changzhou from January 2015 to August 2015 were randomly divided into an sFNB + laryngeal mask airway (FLA) group, an sFNB + tracheal intubation (FGA) group and a tracheal intubation (GA) group. Their clinical parameters and indices were recorded. They were scored by the Visual Analogue Scale (VAS).

**Results:**

All patients completed this study. FLA and FGA groups used less propofol, remifentanil and fentanyl than GA group (P<0.01), with shorter recovery time and extubation time (P<0.05). Compared to GA group, FLA and FGA groups had lower systolic blood pressures at T3, T4 and T5 (P<0.05), and lower heart rates at T5 (P<0.05). FLA and FGA groups had fewer cases of adverse reactions after extubation (P<0.01). FLA group was less prone to irritating cough after extubation and pharyngeal pain than FGA and GA groups (P<0.01). The postoperative six hour and 24 hour VAS scores in resting state as well as the postoperative 24 hour and 48 hour scores in training state of FLA and FGA groups were lower than those of GA group (P<0.05). FLA and FGA groups used significantly lower times and total doses of patient-controlled intravenous analgesia pump.

**Conclusion:**

sFNB combined with general anesthesia, especially that using laryngeal mask, were superior to general anesthesia alone, which reduced recovery and extubation times, and decreased intraoperative and postoperative drug uses, postoperative early VAS score and adverse reactions.

## INTRODUCTION

Currently, the total number of people aged 60 and above has reached about 0.178 billion in China,^1^ ranking first in the world. Primary osteoarthritis (OA) is a common joint degenerative disease, to which the elderly is most vulnerable. Increase of age is positively related with OA incidence. Of the elderly aged over 75, those with symptomatic knee joint OA accounted for approximately 50%.^2^ Total knee arthroplasty (TKA) has been performed in clinical practice since the 1960s,^3^ as a mature therapy now. It can effectively relieve pain and recover joint functions, thus having become the first choice for treating end-stage knee joint OA.

For TKA, nerves of lower limbs, including lumbar plexus-sciatic nerve and femoral-sciatic nerve, are mostly blocked.^4^ However, the lumbar plexus and sciatic nerve are located deeply, so patients should be kept in the lateral position and suffer from long surgical time even if nerve stimulator or ultrasonic assistance is used. In contrast, since the femoral nerve is located shallowly, patients can take the supine position and nerve stimulator or ultrasound allows rapid localization. Besides, nociceptive fiber conduction in front of and inside the knee joint can be effectively blocked after drug infusion. Notably, single femoral nerve block (sFNB) has been highlighted as a postoperative analgesic method.^5^,^6^

Regardless, sFNB should be combined with general anesthesia to achieve ideal anesthesia. In addition, this strategy can improve perioperative comfort, without delaying the postoperative use of anticoagulants. In this study, we evaluated the clinical effects of sFNB in combination with general anesthesia on geriatric patients receiving unilateral total knee arthroplasty (UTKA).

## METHODS

This study has been approved by the ethics committee of our hospital, and written consent has been obtained from all patients. Sixty geriatric UTKA patients who were treated in The First People's Hospital of Changzhou from January 2015 to August 2015 were randomly divided into an sFNB + laryngeal mask airway (FLA) group, an sFNB + tracheal intubation (FGA) group and a tracheal intubation (GA) group (n=20).

### Inclusion criteria

In accordance with OA diagnostic criteria and surgical indications; aged over 60 years. without limitation of gender or surgical sites; ASA (American Society of Anesthesiologists) grade II~III; with written informed consent.

### Exclusion criteria

With anatomic variations in the inguinal region that hindered or affected FNB; with severe cardiovascular diseases, liver or kidney dysfunction, and coagulation dysfunction; allergy to any of tested anesthetic drugs; with history of alcoholism, drug abuse and preoperative use of opioids or sedatives; difficulty in daily communication.

### Anesthesia process: FNB

Before induction of general anesthesia, FLA and FGA groups were subjected to unilateral femoral nerve puncture in the presence of nerve stimulator.

### Surgical methods

In the supine position, lower limbs of patient were slightly separated, and femoral artery was located two cm below the inguinal ligament, one cm from which was given subcutaneous infiltration anesthesia. A 50 mm electric stimulation needle was inserted 1 cm away from the femoral artery in an angle of 30°. The initial stimulation current was one mA which was decreased to 0.3 mA upon obvious rectus femoris contraction or knee jerking. When the rectus femoris was still apparently contracting, 20 mL of 0.375% ropivacaine was slowly injected, during which whether blood return occurred was observed by repeated withdrawal.

### Anesthesia induction

Three groups were intravenously injected with 0.03 mg/kg midazolam, 3-5 μg/kg fentanyl and 1.0-1.5 mg/kg propofol. When they became unconscious and BIS ≤60, 0.6 mg/kg rocuronium bromide was intravenously injected. After 1.5 minutes, the FLA group was inserted 3^#^-5^#^ supreme laryngeal masks according to body weights. FGA and GA groups were endotracheally intubated using 7.5^#^ catheter for males and 7.0^#^ catheter for females. After auscultation suggested clear respiratory sounds in both lungs, an anesthesia machine was connected and mechanical ventilation was performed.

### Anesthesia maintenance

With the oxygen flow of 2 L/min, patients continuously inhaled 0.8% sevoflurane, and were continuously intravenously pump-infused propofol and remifentanil. According to BIS and blood pressure, the infusion rates of propofol and remifentanil as well as the times of using fentanyl additionally were adjusted. Sevoflurane was no longer used during joint capsule suturing, and pump infusion was stopped during skin suturing. During surgery, BIS was maintained at 40-60, the tidal volume was set at 6-8 mL/kg, and the end-tidal carbon dioxide partial pressure was kept at 30-35 mmHg by adjusting respiration-related parameters.

### Intervention measures

During induction and maintenance periods, 0.5 mg atropine or 10 mg esmolol was intravenously injected if heart rate (HR) decreased (<50 bpm) or increased (>110 bpm). If systolic blood pressure (SBP) <90 mmHg or >160 mmHg, 6 mg ephedrine or 10 mg urapidil was intravenously injected. The cases with unsatisfactory outcomes after single administration were then repeatedly administered until vital signs became stable.

### Extubation indications

In the case of extubation indications including spontaneous respiration, recovery of swallowing reflex and muscle strength, responsiveness to calling and Sp O_2_ >90% after three minutes of air inhalation, laryngeal mask was directly removed from the FLA group, and tracheal catheters were extubated from FGA and GA groups after sputum aspiration and lung inflation.

### Postoperative analgesia

Before leaving the operating room, three groups were intravenously given analgesic pump using 0.02 μg/kg sufentanil, 24 mg ondansetron and 100 mL of normal saline. The background infusion rate was 1 mL/h, the single dose was 1 mL, and the lockout time was 15 min. The three groups were continuously analgesized for 72 hour. If the Visual Analogue Scale (VAS) score exceeded four points, 40 mg parecoxib sodium was intravenously infused additionally, twice/day.

### Monitoring indices: Baseline data

Age, gender, body mass index (BMI), surgical time and tourniquet use time of three groups were recorded.

### Intraoperative monitoring indices

HR, SBP and diastolic blood pressure (DBP) were observed before anesthesia (T0), immediately after tracheal intubation or laryngeal mask insertion (T1), at the beginning of surgery (T2), five minutes after tourniquet use (T3), five minutes after stopping using tourniquet (T4) and extubation of tracheal catheter or removal of laryngeal mask (T5). Intraoperative doses of propofol, remifentanil and fentanyl, anesthesia recovery time, extubation time and perioperative use of vasoactive drugs were recorded.

### Postoperative monitoring indices

VAS scores in the resting state of patients 6 hour, 24 hour, 48 hour and 72 hour after surgery and those in the training state 24 hour 48 hour and 72 hour after surgery were recorded. The use of patient-controlled intravenous analgesia (PCIA) pump and parecoxib sodium was recorded. Postoperative adverse reactions such as nausea, vomiting, and urinary retention, hypersomnia, irritating cough and pharyngeal pain were observed.

### Statistical analysis

All data were analyzed by SPSS19.0. The categorical data conforming to normal distribution were expressed as mean ± standard deviation (x ± s), and those not were expressed as median (interquartile range). All data were subjected to normal distribution and homogeneity tests, and the eligible categorical data were subjected to analysis of variance. Otherwise, they underwent the nonparametric rank sum test. The numerical data were given the χ^2^ test. P<0.05 was considered statistically significant.

## RESULTS

### Baseline clinical data

The three groups had similar gender ratio, age, BMI, surgical time and tourniquet use time (P>0.05) (Table-I).

**Table-I:** Baseline clinical data (χ̄ ± s, n=20).

Group	Age (year)	Gender (Male/female)	BMI (kg/m^2^)	Tourniquet use time (min)	Surgical time (min)
FLA	70.4±4.4	17/3	26.6±34	63.3±17.3	98.5±19.3
FGA	72.4±4.2	16/4	26.5±2.0	59.9±10.7	92.0±11.4
GA	71.4±3.7	15/5	27.5±3.2	65.4±17.8	100.3±21.0

### Hemodynamics changes

After induction, SBP, DBP and HP of three groups all decreased, and then basically recovered to normal during surgery. Compared to GA group, FLA and FGA groups had significantly lower SBP values at T3, T4 and T5 (P<0.05), and significantly lower HR values at T5 (P<0.05) (Table-II).

**Table-II:** Hemodynamics changes (χ̄ ±s, n=20).

Index	Group	T0	T1	T2	T3	T4	T5
SBP (mmHg)	FLA	146.2±14.7	120.8±9.8	114.9±8.7	115.1±7.4*	115.0±8.7*	125.0±5.1*
	FGA	140.8±14.0	120.7±9.4	111.1±9.1	111.1±8.4*	116.5±7.6*	125.5±8.9*
	GA	147.5±13.9	125.8±13.2	117.7±12.1	120.6±11.4	123.4±10.9	143.4±21.3
DBP (mmHg)	FLA	76.9±9.0	65.2±6.9	63.4±5.4	62.7±7.1	63.1±9.2	68.3±6.8*
	FGA	74.1±10.7	67.3±7.1	61.7±5.1	64.3±5.5	65.4±7.0	69.8±7.8*
	GA	74.0±7.2	66.9±8.4	63.1±7.9	63.4±8.0	64.4±10.0	77.1±6.2
HR (bpm)	FLA	77.0±11.7	67.7±10.5	64.6±7.7	64.1±8.6	64.5±8.4	70.5±4.6*▲
	FGA	80.4±10.2	71.3±7.6	65.5±7.0	65.9±4.7	65.2±7.7	77.0±4.8*
	GA	78.3±13.6	72.0±8.9	66.9±8.6	66.7±7.7	69.2±16.3	83.3±6.3

Compared with GA group,

* P<0.05; compared with FGA group,

▲ P<0.05.

### Dose of anesthetics, recovery time and extubation time

FLA and FGA groups used significantly less propofol, remifentanil and fentanyl than GA group did (P<0.01), with significantly shorter recovery time and extubation time (P<0.05) (Table-III).

**Table-III:** Dose of anesthetics (*x̄* ±s, n=20).

Group	Propofol (mg)	Remifentanil (mg)	Fentanyl (mg)	Recovery time (min)	Extubation time (min)
FLA	274±31**	0.98±0.27**	0.21±0.08**	6.2±2.1**	8.9±3.2**
FGA	287±29**	1.03±0.26**	0.24±0.07**	6.9±2.2**	10.2±2.6**
GA	395±41	1.34±0.32	0.37±0.07	12.0±3.8	16.8±4.8

Compared with GA group,

** P<0.01.

### Perioperative adverse events and complications

FLA and FGA groups had significantly fewer cases of adverse events and lower incidence rates of complications during surgery (P<0.05). FLA group was significantly less prone to irritating cough after extubation and pharyngeal pain than FGA and GA groups (P<0.05) (Table-IV).

**Table-IV:** Perioperative adverse events and complications (χ̄ ±s, n=20)

Group	Irritating cough after extubation (Case No.)	Restlessness in recovery from anesthesia (Case No.)	Incision pain after extubation (Case No.)	Postoperative pharyngeal pain (Case No.)
FLA	0*▲	0**	0**	0**▲
FGA	5	2**	0**	7
GA	7	8	5	8

Compared with GA group,

* P<0.05,

** P<0.01; compared with FGA group,

▲ P<0.05.

### Postoperative pain

The postoperative 6 h and 24 h VAS scores in the resting state as well as the postoperative 24 hour and 48 hour scores in the training state of FLA and FGA groups were significantly lower than those of GA group (P<0.05). VAS scores in resting and training states of the three groups were similar 72 h after surgery (Table-V).

**Table-V:** VAS scores in resting and training states (M (X25-X75), n=20).

	FLA group	FGA group	GA group
VAS score in resting state		
6 h	2 (1.75-3.0)*	2 (1.75-3)*	3 (3-4)
24 h	2 (1-3)*	2.5 (2-3)*	4 (3-4.25)
48 h	1 (1-2)	2 (1-2.25)	2 (1-3)
72 h			
VAS score in training state		
24 h	3 (2-3.25)*	3 (2-4)*	4.5 (4-5.25)
48 h	2.5 (2-3)*	3 (2-3)*	4 (3-4.25)
72 h	4 (3-4.25)	2 (1-2)	2 (1.75-2.25)

Compared with GA group,

* P<0.05.

### Use of analgesics

FLA and FGA groups used significantly lower times and total doses of PCIA pump, and doses of parecoxib sodium used additionally (P<0.01). There were no significant differences between FLA and FGA groups (P>0.05) (Table-VI).

**Table-VI:** Postoperative use of PCIA pump and other analgesics (χ̄ ±s, n=20).

Group	Pressing of PCIA pump (time)	Total dose of PCIA pump (mL)	Parecoxib sodium (mg)
FLA	3.7±1.1**	75.8±2.0**	32.0±12.1**
FGA	3.2±1.0**	75.6±1.3**	30.0±12.4**
GA	7.3±1.5	79.2±1.6	136.0±

Compared with GA group,

** P<0.01.

### Postoperative adverse reactions

The incidence rates of postoperative adverse reactions were similar (P>0.05). No patient discontinued using analgesics due to adverse reactions.

## DISCUSSION

The proportion of elderly population has increased annually worldwide. TKA has been widely used in clinical practice to treat geriatric patients with knee joint diseases. However, these patients are often complicated with cerebrovascular, cardiovascular and respiratory diseases. During surgery, they have low stress response as well as fluctuated heart rate and blood pressure, failing to maintain stable hemodynamics or to tolerate anesthesia.^7^ Thus, it is necessary to select a safe, effective anesthetic method with obvious outcomes, mild side effects and slight interference with respiratory cycle. Currently, lower limb surgeries are mainly anesthetized by general anesthesia, nerve block and intravertebral anesthesia.^8^ Although general anesthesia works quickly and allows easy intraoperative management, the postoperative recovery and quality of life of geriatric patients may be severely affected due to considerable changes of hemodynamics, increased sensitivity to anesthetics, decelerated metabolism and cardiovascular stress response.

In recent years, nerve stimulation- and ultrasound-guided assisted puncture has greatly improved the success rate of nerve block,^9^ which has become popular in the lower limb surgery on elderly patients because of its small impact on circulation and respiration.^10^ Good lower limb nerve block can not only provide the same analgesic effect as epidural anesthesia, reduce the dose of fentanyl and other opioids, but also lower the incidence of complications.^11^-^13^ Compared with simple general anesthesia, this study used sFNB combined general anesthesia, which had better perianesthesia cardiovascular stability and less hemodynamic fluctuations. After tightening and loosing tourniquet, SBP of the simple general anesthesia group (GA group) was significantly higher than those in the FNB combined general anesthesia group (FLA group and FGA group), but there was no significant difference between FLA group and FGA group, which might because simple general anesthesia cannot completely block the noxious stimulation caused by tourniquet. It has been reported that the incidence of tourniquet pain were 2.5%, 2.7% and 67% after brachial plexus block, spinal anesthesia and general anesthesia, respectively.^14^ FNB combined general anesthesia can reduce the doses of intraoperative narcotics and sedative analgesic drugs, shorten the recovery time and extubation time of elderly patients, and further improve the safety of UTKA perioperative anesthesia management.^15^ In addition, the hemodynamics during the anesthesia induction and recovery period is more stable, while effectively avoiding the adverse effects of general anesthesia endotracheal intubation on the body and reducing perioperative complications,^16^ which is significantly better than general anesthesia endotracheal intubation.

Multi-mode analgesia can exert synergistic analgesic effects to reduce the amounts of different analgesic drugs.^17^ For FNB, sufficient local anesthetic liquid with low concentrations needs to be injected preoperatively to pre-block or reduce the nociceptive transmission at the two stages of surgical injury and inflammatory response, so as to alleviate the central sensitization and achieve good effects of postoperative analgesia.^18^ In this study, patients of each group were treated with PCIA analgesia, and the VAS score of the FLA group and the FGA group was significantly lower than that of the GA group 6 h to 24 h after surgery, which might be associated with the slowdown of local anesthetic metabolism in the elderly patients, so that the duration of using analgesics was prolonged. The patients had gone through the acute pain period 48 h after surgery, and then the VAS score of resting state was less than 3 points in all the three groups, showing mild pain. Hence, sFNB combined general anesthesia had a better postoperative analgesic effect on the early postoperative acute pain, being consistent with a previous literature.^19^

### Limitations of the study.

The risk of infection would be substantially increased for elderly patients with continuous femoral nerve block time of over 48 h as the femoral nerve puncture catheter was near the perineum, with potential risk of infection, and the patients were often associated with diabetes preoperatively.^20^ In addition, TKA patients were often prescribed with low molecular weight heparin and other anticoagulants before and after surgery to prevent the occurrence of deep vein thrombosis, so there is a potential risk of bleeding at the puncture site, and catheterization may increase the risk of local hematoma formation. It is difficult to fix the femoral nerve catheterization, and especially the postoperative early activities of patients increase the risk of catheter detachment. Based on the above considerations, this study used sFNB, without carrying out control study on the postoperative analgesic evaluation of cFNB and sFNB.

## CONCLUSION

In summary, sFNB combined general anesthesia for elderly patients, general anesthesia of laryngeal mask in particular, exerted evident intraoperative and postoperative analgesic effects. This strategy reduced both perioperative use of anesthetics and the incidence rates of related complications. It is safer and more effective for elderly patients. Further studies using the knee flexion angle test are ongoing in our group.

### Authors' Contributions

**JZ:** Study design and manuscript preparation.

**YY, YZ & YW:** Clinical data collection and analysis.
